# Contribution of common and rare damaging variants in familial forms of bipolar disorder and phenotypic outcome

**DOI:** 10.1038/s41398-020-0783-0

**Published:** 2020-04-28

**Authors:** Elisa Courtois, Mark Schmid, Orly Wajsbrot, Caroline Barau, Philippe Le Corvoisier, Bruno Aouizerate, Frank Bellivier, Raoul Belzeaux, Caroline Dubertret, Jean-Pierre Kahn, Marion Leboyer, Emilie Olie, Christine Passerieux, Mircea Polosan, Bruno Etain, Stéphane Jamain, and the FondaMental Advanced Centers of Expertise in Bipolar Disorders (FACE-BD)

**Affiliations:** 1 INSERM U955, Psychiatrie Translationnelle, Créteil, 94000 France; 2 Université Paris Est, Faculté de Médecine, Créteil, 94000 France; 3 Fondation FondaMental, Créteil, 94000 France; 4 Université de Lorraine, CHRU de Nancy et Pôle de Psychiatrie et Psychologie Clinique, Centre Psychothérapique de Nancy, Laxou, 54520 France; 5 AP-HP, Hôpital H. Mondor–A. Chenevier, Plateforme de Ressources Biologiques, Créteil, 94000 France; 6 Inserm, Centre d’Investigation Clinique 1430 and APHP, Henri Mondor Hospital, Créteil, 94000 France; 7 Centre Expert Troubles Bipolaires, Service de Psychiatrie Adulte, Hôpital Charles-Perrens, Bordeaux, 33000 France; 8 AP-HP, GH Saint-Louis-Lariboisière-F. Widal, Département de Psychiatrie et de Médecine Addictologique, Paris, 75010 France; 9 Université Paris Diderot, Sorbonne Paris Cité, Paris, 75010 France; 10 Inserm, UMR-S1144, Paris, 75010 France; 11 Pôle de Psychiatrie, Assistance Publique Hôpitaux de Marseille, INT-UMR7289, CNRS Aix-Marseille Université, Marseille, 13009 France; 12 AP-HP, Département de Psychiatrie, Hôpital Louis Mourier, INSERM U894, Université de Paris, Colombes, 92700 France; 13 AP-HP, DHU PePSY, Pôle de Psychiatrie et d’Addictologie des Hôpitaux Universitaires Henri Mondor, Créteil, 94000 France; 14 Département urgence et Post-urgence psychiatrique, CHU Montpellier, INSERM U1061, Université de Montpellier, Montpellier, 34000 France; 15 Service Universitaire de Psychiatrie d’Adultes, Centre Hospitalier de Versailles, Laboratoire HandiRESP, EA4047, UFR des Sciences de la Santé Simone Veil, Université de Versailles Saint-Quentin-En-Yvelines, Le Chesnay, 78150 France; 16 Université Grenoble Alpes, CHU de Grenoble et des Alpes, Grenoble Institut des Neurosciences (GIN) Inserm U 1216, La Tronche, 38700 France

**Keywords:** Comparative genomics, Clinical genetics, Bipolar disorder

## Abstract

Genome-wide association studies on bipolar disorders (BD) have revealed an additive polygenic contribution of common single-nucleotide polymorphisms (SNPs). However, these SNPs explain only 25% of the overall genetic variance and suggest a role of rare variants in BD vulnerability. Here, we combined high-throughput genotyping data and whole-exome sequencing in cohorts of individuals with BD as well as in multiplex families with a high density of affected individuals in order to determine the contribution of both common and rare variants to BD genetic vulnerability. Using polygenic risk scores (PRS), we showed a strong contribution of common polymorphisms previously associated with BD and schizophrenia (SZ) and noticed that those specifically associated with SZ contributed more in familial forms of BD than in non-familial ones. The analysis of rare damaging variants shared by affected individuals in multiplex families with BD revealed a single interaction network enriched in neuronal and developmental biological pathways, as well as in the regulation of gene expression. We identified four genes with a higher mutation rate in individuals with BD than in the general population and showed that mutations in two of them were associated with specific clinical manifestations. In addition, we showed a significant negative correlation between PRS and the number of rare damaging variants specifically in unaffected individuals of multiplex families. Altogether, our results suggest that common and rare genetic variants both contribute to the familial aggregation of BD and this genetic architecture may explain the heterogeneity of clinical manifestations in multiplex families.

## Introduction

Bipolar disorders (BD) are chronic, heterogeneous, and complex mental disorders with a worldwide prevalence of ~1%^1^. Their etiopathology is poorly understood but genetic contribution arises has an important factor, with an estimated heritability ranging between 60% and 80%^2,3^. Recent genome-wide association studies (GWAS) have demonstrated the contribution of common variants in the increased vulnerability of developing BD^4^. Associated variants had a low effect size, but when combined in an additive model, they were able to significantly distinguish individuals with BD from controls^5^. However, these overall results explain only 25% of the genetic variance^6^, revealing a “missing heritability” and increasing the potential interest for rare penetrant variants.

The rapid development of next-generation sequencing led to the possibility to unravel the potential contribution of rare variants in BD. In other psychiatric disorders, e.g. autism spectrum disorder (ASD) and schizophrenia (SZ), whole-exome sequencing (WES) has already shown a higher frequency of de novo mutations in affected individuals compared to control populations^7,8^. However, few studies have been conducted yet in subjects with BD with a limited consensus on rare penetrant mutation burden and their relationship with low penetrant common variants^9–15^.

Here, we combined high-throughput genotyping data and WES in cohorts of individuals with BD as well as in multiplex families with a high density of affected individuals in order to determine the relative contribution of common and rare variants in clinical manifestations of BD. We first calculated polygenic risk scores (PRS) for BD and SZ in a cohort of 445 patients with BD and 1636 individuals from the general population, and determine their impact on the family history of BD. We then used these scores in multiplex families and compared affected and unaffected individuals. Using WES, we identified rare damaging variants in constrained genes that were shared by affected subjects and highlighted impacted biological pathways. Association with BD was confirmed by comparing mutation rates of associated genes in an independent cohort of 241 subjects with BD and 21,071 non-Finnish European individuals from the non-psychiatric population of the Exome Aggregation Consortium (ExAC)^16^. Finally, we combined PRS and rare variants identified in families and showed different genetic architectures between affected and unaffected individuals in multiplex families.

## Materials and methods

### Multiplex families

A written informed consent was obtained from all subjects prior to participation in the study and research ethic board of the Pitié-Salpêtrière Hospital (CPP Ile de France VI) approved protocols and procedures.

We recruited eight multiplex families (Supplementary Fig. S1) of French ancestry with at least two individuals meeting the BD criteria of the fourth edition of the diagnostic and statistical manual for psychiatric disorders (DSM-IV)^17^. In total, we collected DNA for 38 individuals: 21 with BD, 1 with schizo-affective disorder (SAD), 1 with ASD, 1 with unipolar depression, 1 with major depressive episode, and 13 healthy individuals. Taking into account the high shared heritability between BD and SZ and to avoid too much heterogeneity, we considered, as “affected” subjects in multiplex families, those with a diagnosis of either a BD or a SAD. All other phenotypes and unaffected subjects were pooled under the name “unaffected”.

### Cohorts

Two-hundred-and-eighty patients with BD were recruited in nine FondaMental Academic Centers of Expertise for Bipolar Disorder (FACE-BD) located in France^18^. This network is coordinated by the French scientific cooperation foundation, Fondation FondaMental (www.fondation-fondamental.org). All patients met the DSM-IV diagnostic criteria for BD. In addition, a previously published cohort^19,20^ of 445 subjects with BD and 1636 control individuals of French origin has been used for genotyping analyses (Supplementary Fig. S2).

### DNA extraction and genotyping

Genomic DNA was isolated from venous blood sample or from saliva. DNA extraction was performed as previously described^21^ for blood samples or using prepIT® L2P kit (DNA genotek, Kanata, ON, Canada), following the manufacturer’s instructions. In multiplex families, 27 DNAs were genotyped for ~960,000 single-nucleotide polymorphisms (SNPs) using the OmniExpressExome-8v1-4_A1 beadchips (Illumina Inc., San Diego, CA, USA) and 11 DNAs were genotyped for ~660,000 SNPs, using the Infinium Global Screening Arrays-24 v2.0 (Illumina Inc.), according to the manufacturer’s protocol (Supplementary Fig. S2). Quality control of genotypic data, family relationships, and ancestry were performed using the PLINK (v.1.9)^22^, the KING (v.2.1.3)^23^, and the Eigensoft (v.7.2.1)^24^ softwares, respectively. Briefly, samples with sex discordance, a heterozygosity rate different of the mean_het_ ± 0.2, a genotyping rate lower than 0.98 or without a European ancestry were excluded of analyses. SNPs with a genotyping rate lower than 0.95 were removed. Remaining SNPs were subsequently used for imputation in 37 samples using the Sanger imputation server. Prephasing was conducted using SHAPIT2 (v2.r790)^25^ and imputation using PBWT^26^ with 1000 Genomes phase 3 as reference panels^27^. After imputation, only biallelic SNPs with a minor allele frequency (MAF) higher than 0.01 in the reference panel and an imputation score higher than 0.9 were used for further studies.

The previously published cohort has been genotyped using HumanHap550 bead arrays (Illumina Inc.) for individuals with BD and HumanHap300 bead arrays (Illumina Inc.) for controls and imputed by the Psychiatric Genomics Consortium (PGC) based on HapMap 2 CEU sample^19^.

### Polygenic risk score analysis

PRS were calculated for each individual using the PRSice software (v.2.0.15)^28^ and were based on the PGC summary statistics for BD^4^ or SZ^29^ with only European individuals. The previously published cohort was included in the PGC BD summary statistics. Therefore, summary statistics excluding the French cohort have been generously provided by Dr. Eli Stahl, who conducted the last PGC BD analyses. Only genotyped or imputed independent SNPs (*r*^2^ < 0.2) both in multiplex families and in the previously published cohort with a MAF higher than 0.01 and an imputation score higher than 0.9 were used for PRS calculation. We estimated the best PRS threshold to discriminate patients from controls in our cohort by testing all *p* values threshold between 0.00001 and 0.5 with an incremental factor of 0.00001. We then selected the one showing the lowest *p* value and the highest *R*^2^. We obtained a *p* value threshold of 0.3319 for the BD-associated SNPs-based PRS (BD-PRS) and of 0.2025 for SZ-associated SNPs-based PRS (SZ-PRS) (Supplementary Figs. S3 and S4, respectively). In addition, at the threshold previously determined, we isolated SNPs that only contributed to BD-PRS (BD_only_-PRS), i.e. only present in BD summary statistics with INFO > 0.9 and *p* value < 0.3319, from those that only contributed to SZ-PRS (SZ_only_-PRS), i.e. only present in SZ summary statistic with INFO > 0.9 and *p* value < 0.2025, and from those that contributed both to BD and SZ, i.e. present in BD summary statistics with INFO > 0.9 and *p* value < 0.3319 and SZ summary statistics with INFO > 0.9 and *p* value < 0.2025. For the latter ones, we calculated a new PRS (BD–SZ-PRS) based on the average effect, i.e. the mean of the odds ratio (OR), observed in the SZ and the BD summary statistics. The correlation between PRS and the number of rare damaging variants in multiplex families was calculated using the sum of the scores (PRS_sum_) of BD_only_-PRS, SZ_only_-PRS, and the BD–SZ-PRS for each individual.

### Exome sequencing

Exons were captured using the SureSelect^XT^ Human All Exon V5, the SureSelect^QXT^ Human All Exon V6 or the SureSelect^QXT^ Human All Exon V6+ UTR libraries (Agilent Technologies, Santa Clara, CA, U.S.A.). WES was conducted on NextSeq500 with paired-end 75 bp reads, HiSeq2000 with paired-end 75 bp reads, or HiSeq2500 with paired-end 125 bp reads. Data processing consisted in using an home-made workflow based on Trimmomatic (v.0.36)^30^, BWA-MEM (v.0.7.12)^31^, Picard (v.2.8.1), and GATK (v.3.7)^32^ softwares. A bed intersect file between the three different captures generated with BedOps (v.2.4.28)^33^ and the human reference genome (hg19:GRCh37) were used. Variant calling was performed following the GATK best practices recommendations and annotation was made with SnpEff (v.4.3)^34^ and bcftools (v1.6). Only variants passing quality filter of VariantRecalibration tool of GATK, with a depth of read higher than 10, a genotype quality score higher than 20, and no missingness in family were kept. Same quality control of individuals as for beadchips was applied using the PLINK, KING, and Eigensoft softwares except a genotyping rate lower than 0.94. After quality control, 34 samples were used in subsequent analysis. In order to identify relevant variants, we selected variants absent from the ExAC^16^ or the 1000 Genomes^27^ databases or with a MAF lower than 0.01 in these two databases. We then selected damaging variants, i.e. missense variants, indels, frameshift, loss or gain of stop or start codon, and those altering splice sites, with a phred CADD score higher than 15 (v1.4)^35^, corresponding to the 5% most deleterious variants in the genome. We also selected variants in constrained genes, i.e. with a *z*-score higher than 3 for missense variants and inframe indels, or with a pLI > 0.9 for loss-of-function variants^16^. All the mutations identified have been checked by Sanger sequencing.

For the cohort of 280 subjects with BD, the same quality control was performed. In addition, related individuals and variants with a missingness higher than 0.03 were excluded. After quality control, 241 were used in subsequent analyses (Supplementary Fig. S2).

For general population, we downloaded vcf files (ftp://ftp.broadinstitute.org/pub/ExAC_release/release1/subsets/ExAC_nonpsych.r1.sites.vep.vcf.gz) corresponding to non-psychiatric individuals of the ExAC population^16^ and selected only non-Finnish European individuals. We then extracted damaging variants (missense or loss-of-function) with an MAF < 1% and which passed filter of VariantRecalibration tool of GATK in mutated genes identified in our multiplex families. Only variants covered at 95% of the maximum count of allele were considered and only genes with more than 70% of their variants meeting this criterion were considered.

### Network and pathway analyses

Network and pathway analyses were performed using the R version of the STRING (v.10) database^36^. Network was defined using a minimum interaction score of 0.4 and with active interaction sources as text-mining, co-expression, experiments, and database. Pathway analyses were based on the Gene Ontology (GO) resource^37,38^ and a custom background gene list of 18,016 genes. Only GO term including between 5 and 1800 genes and pathways with at least 10% of mutated genes were considered for enrichment analyses.

Gene length bias was controlled by randomly selecting 10,000 times the number of genes identified in multiplex families (*N* = 34) with a cumulative exon length equal to the mean size of mutated genes (±20%). GO terms were removed when they were found more than 500 times after 10,000 permutations.

### Statistical analyses

The data that support the findings of this study are available from the corresponding author upon reasonable request.

Statistical analyses were conducted with R (v.3.4.1). All variables were expressed as median and interquartile range and between-group comparisons were analyzed by Wilcoxon rank-sum tests. Group comparison proportions were analyzed using the Fisher’s exact test or Chi-squared test according to sample size. Benjamini–Hochberg correction was applied for multiple testing when comparing mutation rates. Differences were considered as significant when the false discovery rate (FDR) was lower than 0.05. Bonferroni correction was applied for PRS comparisons, multivariate PRS analyses, clinical data analyses, and linear regression between PRS_sum_ and the number of rare mutations. In all cases, *p* values were adjusted (*p*_adj_) according to the number of tests, i.e. 12, 3, 5, and 2, respectively, and difference were considered as significant when the *p*_adj_ was lower than 0.05.

## Results

### SZ-specifically associated SNPs contribute only to familial forms of BD

**Fig. 1:**
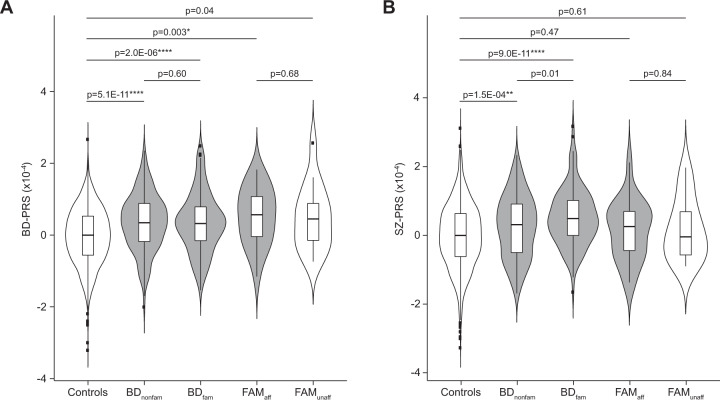
Polygenic risk scores for bipolar disorder and schizophrenia in multiplex families and the general population. Violin plot showing polygenic risk scores for bipolar disorder (BD-PRS) (**a**) and schizophrenia (SZ-PRS) (**b**) calculated for affected (gray) and unaffected (blank) individuals. Scores were normalized on the control median value and compared between controls (*N* = 1636), non-familial forms of bipolar disorder (BD_nonfam_, *N* = 218), familial forms of bipolar disorder (BD_fam_, *N* = 144), affected subjects in multiplex families (FAM_aff_, *N* = 21), and unaffected subjects in multiplex families (FAM_unaff_, *N* = 16) with Wilcoxon test. A *p* value threshold of 0.3319 including 27,100 independent SNPs was chosen for BD-PRS as the best threshold to discriminate between individuals with bipolar disorder (BD) and controls. A *p* value threshold of 0.2025 including 21,668 independent SNPs was chosen for SZ-PRS as the best threshold to discriminate BD and controls. Nominal *p* values are indicated for each comparison. Significant results after Bonferroni correction for multiple testing are indicated with stars: *p_adj_ < 0.05, **p_adj_ < 0.01, ***p_adj_ < 0.001, ****p_adj_ < 0.0001.

Common polymorphisms have been demonstrated to explain up to 25% of the genetic variance in BD, more than half of which could be shared with SZ^6^. In order to determine whether the polygenic contribution was similar in familial and non-familial forms of BD, we used imputed genotyping data of 1,709,567 SNPs for 445 patients with BD, 1636 controls, and 37 individuals in multiplex families to calculate two PRS based on PGC BD^4^ and SZ^29^ summary statistics. Both BD-PRS and SZ-PRS contributed to BD vulnerability for subjects with (*N* = 144) and without (*N* = 218) a family history of BD when compared with controls (Fig. 1). Although there was no difference in BD-PRS between individuals with and without a family history of BD in our cohort (Wilcoxon rank-sum test, *W* = 16,212; *p* = 0.60; *p*_adj_ = 1.00; Fig. 1a), a higher SZ-PRS was observed for familial forms when compared to non-familial ones (Wilcoxon rank-sum test, *W* = 13,225; *p* = 0.01; *p*_adj_ = 0.12; Fig. 1b). However, this difference did not resist to correction for multiple testing. We then split PRS with the aim of distinguishing SNPs that were specifically associated with BD (BD_only_-PRS) or SZ (SZ_only_-PRS), and those that contributed to both disorders (BD–SZ-PRS) and performed multivariate analyses (Table 1). While the SZ_only_-PRS and the BD–SZ-PRS both contributed to the risk of developing BD in familial forms of the disease, only the BD-associated polymorphisms (BD_only_-PRS and BD-SZ-PRS) contributed to the disease vulnerability in non-familial forms of BD. Consistent with this observation, we showed that SZ specific associated polymorphisms (SZ_only_-PRS) were able to distinguish familial and non-familial forms of BD (Table 1).

In multiplex families, we observed that affected subjects had a higher BD-PRS than controls (Wilcoxon rank-sum test, *W* = 10,675; *p* = 0.003, *p*_adj_ = 0.04; Fig. 1a). Although, BD-PRS was slightly higher in unaffected subjects in multiplex families, this difference did not resist to correction for multiple testing (Wilcoxon rank-sum test, *W* = 9199; *p* = 0.04, *p*_adj_ = 0.48) and no difference was observed between affected and unaffected subjects (Wilcoxon rank-sum test, *W* = 182; *p* = 0.68, *p*_adj_ = 1.00). In contrast, no higher SZ-PRS was observed neither for affected (Wilcoxon rank-sum test, *W* = 15,589, *p* = 0.47, *p*_adj_ = 1.00) nor for unaffected (Wilcoxon rank-sum test, *W* = 12,124, *p* = 0.61, *p*_adj_ = 1.00; Fig. 1b) subjects in multiplex families. These results suggested that common polymorphisms were unable to explain alone the clinical status differences observed in multiplex families of BD.

**Table 1:** Multivariate analyses in family and non-familial forms of bipolar disorder using polygenic risk scores based on bipolar disorder-specific-, schizophrenia-specific-, and bipolar disorder and schizophrenia shared-associated variants.

	*β*	s.e.	*z*^a^	*p*	*p*_adj_^b^
BD_fam_ vs. controls					
BD_only_-PRS	2477.5	1097.9	1.64	0.02	0.07
** SZ**_**only**_**-PRS**	**2892.4**	**931.6**	**2.26**	**0.002**	**0.006**
** BD-SZ-PRS**	**3624.7**	**739.5**	**4.90**	**9.5E-07**	**2.9E-06**
BD_nonfam_ vs. controls					
** BD**_**only**_**-PRS**	**3022.6**	**934.6**	**3.23**	**0.001**	**0.004**
SZ_only_-PRS	44.7	767.0	0.06	0.95	1
** BD-SZ-PRS**	**3162.6**	**598.6**	**5.28**	**1.3E-07**	**3.8E-07**
BD_fam_ vs. BD_nonfam_					
BD_only_-PRS	−270.2	1362.8	−0.20	0.84	1
** SZ**_**only**_**-PRS**	**3229.7**	**1204.4**	**2.68**	**0.007**	**0.02**
BD-SZ-PRS	384.6	847.0	0.45	0.65	1

*BD* bipolar disorder, *BD*_*fam*_ familial forms of BD, *BD*_*nonfam*_ non-familial forms of BD, *PRS* polygenic risk score, *p* nominal *p* value, *s.e.* standard error, *SZ* schizophrenia.

^a^Wald test.

^b^Adjusted *p* value after Bonferroni correction for multiple testing. Significant results (*p*_adj_ < 0.05) are indicated in bold.

### Rare damaging variants shared by affected subjects in multiplex families affect gene regulation or neuron development and morphogenesis

In order to determine whether rare variants may also contribute to the risk of developing BD in multiplex families, we carried out a WES on 34 subjects from the 8 multiplex families. The mean coverage was 91.5X with 94.7% of bases covered by 20 reads on average. We identified on average 38,347 variants per individual. Focusing on constrained genes has been shown in the past to be a relevant strategy to identify genes associated with multiple psychiatric disorders^10,39^. Thus, after variants filtration, we had on average four rare deleterious variants in constrained genes shared by affected individuals per family (Supplementary Table S1) for a total of 34 mutations located in 34 different genes. We then conducted a network and pathway analysis with STRING software in order to determine whether the proteins encoded by these genes worked in interaction or belonged to the same biological pathways. We showed that genes with mutations have a protein–protein interaction enrichment (*p* = 0.01) (Fig. S5), highlighting their biological connections. The GO terms analysis showed an overrepresentation of 63 biological pathways (FDR < 0.05), and all but three resisted to 10,000 random permutations when we checked for putative gene size bias (Supplementary Table S2). Combining GO data (Supplementary Tables S2–S4) with a review of the literature, we were able to gather functionally convergent biological pathways and showed that the 34 genes were involved either in the development and morphogenesis (*N* = 31), or the regulation of gene expression (*N* = 25), or both (*N* = 22). In addition, we showed that 29 of them were expressed in neurons (Supplementary Fig. S5 and Supplementary Table S3).

### Genes with shared damaging mutation in multiplex families are more frequently mutated in patients with BD than in the general population

In order to determine whether the genes with shared damaging mutations in multiplex families were associated with BD, we sequenced 241 additional independent individuals with BD and compared the mutation rate in patients with the 21,071 non-Finnish European individuals of the ExAC non-psychiatric population. We selected rare (MAF < 1%) missense or loss-of-function variants according to the type of mutations identified in multiplex families in 21 genes. Four genes (*SMARCC2, WDR37, UPF2*, and *HELLS*) showed a higher mutation rate in individuals with BD than in the ExAC population after correction for multiple testing (Table 2).

**Table 2:** Mutation rate in BD cohort and ExAC database for genes mutated in multiplex families.

Gene symbol	Mutation type	Mutation rate in BD^a^ (*N* = 249)	Mutation rate in ExAC cohort^b^ (*N* = 21,071)	*p* value^c^	OR [95% CI]	FDR^d^
*WDR37*	LoF	0.004	0.0000	0.0001	+∞ [24.42; +∞]	0.002
*SMARCC2*	Missense	0.016	0.004	0.0006	4.51 [2.20; +∞]	0.006
*UPF2*	Missense	0.020	0.007	0.003	3.01 [1.61; +∞]	0.02
*HELLS*	Missense	0.018	0.007	0.008	2.72 [1.40; +∞]	0.04
*HSPH1*	LoF	0.002	0.0001	0.06	21.13 [0.87; +∞]	0.21
*SETD2*	Missense	0.028	0.018	0.06	1.6117 [0.96; +∞]	0.21
*ATP2B2*	Missense	0.016	0.010	0.15	1.56 [0.77; +∞]	0.37
*VCL*	Missense	0.014	0.009	0.15	1.64 [0.76; +∞]	0.37
*RBM14*	Missense	0.012	0.007	0.16	1.6 [0.72; +∞]	0.37
*RPTOR*	Missense	0.008	0.005	0.24	1.61 [0.54; +∞]	0.50
*EIF3L*	Missense	0.004	0.002	0.3	1.86 [0.3254; +∞]	0.57

*OR [95% CI]* odds ratio with 95% confidence interval, *LoF* loss-of-function variant.

^a^BD corresponding to 241 individuals of WES cohort +1 proband by family.

^b^Non-Finnish and non-psychiatric European population of ExAC.

^c^One-sided Fisher exact test.

^d^False discovery rate after Benjamini–Hochberg correction for multiple testing.

### Patients with mutations in BD-associated genes display a specific clinical profile

**Fig. 2:**
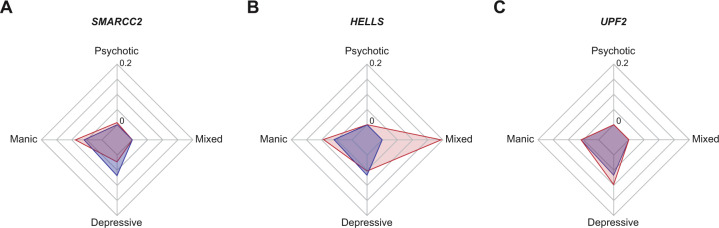
Clinical profiles of mutated individuals in genes with a higher mutation rate in bipolar disorder. Radar plots represent the median number of manic, depressive, or mixed episodes, as well as those with psychotic symptoms for individuals with (red) or without (blue) mutation in *SMARCC2* (**a**), *HELLS* (**b**), and *UPF2* (**c**). For graphical representation, we performed a feature scaling by normalizing data and zoomed on the median values for each feature.

For the four genes with a higher mutation rate in patients with BD than in general population, we analyzed the clinical profile of mutated subjects. As the severity of illness may depend on its duration, we first checked that there was no difference in the duration of illness between subjects with and without mutations in associated genes (Wilcoxon rank-sum test, *W* = 1635, *p* = 0.60, *p*_adj_ = 1.00). In order to determine whether carrying a rare damaging mutation increased the severity of the disease, we calculated a severity score that combined the age at onset, the age at first hospitalization, as well as the number of total episodes and the number of hospitalizations per year of disease. Mutated individuals showed no difference in severity of the disease when compared with non-mutated ones (Wilcoxon rank-sum test, *W* = 1497, *p* = 0.59, *p*_adj_ = 1.00; Supplementary Fig. S6). Nevertheless, we observed particular clinical manifestations for individuals with mutation in some of the BD-associated genes (Fig. 2). Thus, individuals with *SMARCC2* mutations had a predominant manic polarity, displaying more manic than depressive episodes in total (*χ*^2^ = 15.3, df = 1, *p* = 9.1 × 10^−5^, *p*_adj_ = 4.6 × 10^−^^4^; Fig. 2a). Furthermore, individuals with missense variants in *HELLS* displayed more mixed episodes per year of disease than non-mutated subjects (Wilcoxon rank-sum test, *W* = 884, *p* = 0.006, *p*_adj_ = 0.03; Fig. 2b). Although individuals with missense variants in *UPF2* seemed to have more depressive episodes than non-mutated patients, this difference was not significant (Wilcoxon rank-sum test, *W* = 865, *p* = 0.49, *p*_adj_ = 1.00; Fig. 2c). As only two patients had mutation in *WDR37* and only one had available clinical data, we were unable to perform statistical analysis for this gene.

### Affected and unaffected subjects in multiplex families display different genetic pattern of vulnerability

**Fig. 3:**
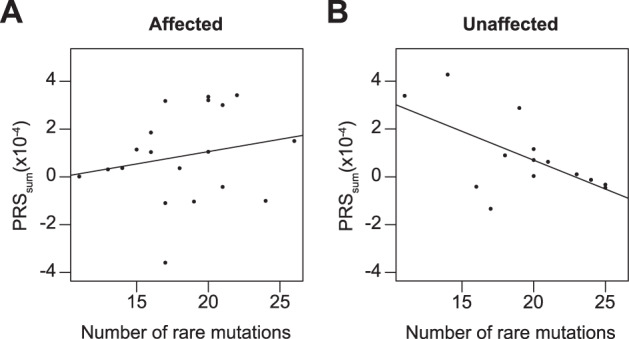
Correlation between the polygenic risk score and the number of rare damaging mutations in multiplex families with bipolar disorder. Each dot represents a single individual and lines symbolize results of the linear regression. The polygenic risk score (PRS_sum_) results from the sum of the scores of BD_only_-PRS, SZ_only_-PRS, and the BD–SZ-PRS for each individual. **a** No correlation was observed between the polygenic risk score and the number of rare damaging mutations in affected individuals (*R*^2^ = −0.014, *F* = 0.76, *p* = 0.40, *p*_adj_ = 0.80). **b** Unaffected individuals showed a significant negative linear regression (*R*^2^ = 0.32, *F* = 7.19, *p* = 0.02, *p*_adj_ = 0.04) between the polygenic risk score and the number of rare damaging mutations.

We showed that BD- and SZ-associated polymorphisms both contributed to familial forms of BD. In addition, we identified rare variants in constrained genes that were shared by affected subjects in multiplex families and which may also contribute to the BD vulnerability. In order to explore the genetic architecture explaining the clinical difference between affected and unaffected individuals in multiplex families, we computed a new PRS (PRS_sum_) combining the information from BD- and SZ-PRS and analyzed this score in regard to the number of rare mutations identified in each individual. Whereas no correlation was observed for affected individuals (*R*^2^ = −0.014, *F* = 0.76, *p* = 0.40, *p*_adj_ = 0.80; Fig. 3a), a negative linear regression was observed between the PRS_sum_ and the number of rare variants in unaffected individuals (*R*^2^ = 0.32, *F* = 7.19, *p* = 0.02, *p*_adj_ = 0.04; Fig. 3b). The observation that only unaffected individuals in multiplex families had either a high PRS_sum_ and low rate of rare damaging mutations or the opposite, suggested the need to reach a threshold of BD-associated variants to declare the disease and showed the important role of both common and rare variants in BD vulnerability.

## Discussion

In this study, we explored the genetic architecture of familial forms of BD combining both common and rare variant analyses. PRS has been demonstrated to be a powerful tool to show the role of common polymorphisms in many psychiatric disorders as well as their shared heritability^6^. However, few studies looked at PRS in families of individuals with BD. Here, we report a higher BD-PRS in families of individuals with BD than in the general population, but no difference was observed between affected and unaffected subjects in multiplex families. Although the higher PRS in BD families is well supported by previous studies^15,40,41^, those calculating a PRS for unaffected subjects reported inconsistent results depending on the number of subjects under study^13,15,41^. Here, the small sample size in multiplex families prevent to exclude any differences between affected and unaffected individuals. Indeed, we estimated that we had a statistical power of 80% to show a large difference (Cohen’s *d* = 0.7) between affected and unaffected individuals, but only 12.5% chance to detect a small effect size (*d* = 0.2). These observations pinpoint the importance of having cohorts as large as possible to conduct such studies and replication on larger samples is required to exclude a difference in PRS between affected and unaffected individuals of multiplex families. Nevertheless, in the whole population of individuals with BD (*N* = 362), we have been able to show that the PRS based on SNPs that have been specifically associated with SZ were higher only in familial forms of BD. The difference in sample size between SZ and BD cohorts to calculate the summary statistics might make the SZ-PRS more robust than the BD-PRS. However, according to the sample size of this cohort, we estimated that we had a statistical power higher than 80% to show a difference in BD-PRS between familial and non-familial forms of BD if this difference was similar to the one observed using SZ-PRS. SZ-PRS has already been shown to explain a shared genetic variance between SZ and BD^5,6^. But difference between familial and non-familial forms of BD had never been reported yet. Thus, our results suggest that the shared genetic vulnerability between SZ and BD might concern mainly familial forms of BD. This is consistent with the observation of a high frequency of individuals with SAD in relatives of individuals with BD^42^. In the multiplex families that we studied, no significant difference between affected and unaffected subjects was detected. Although the small number of individuals in multiplex families limits our possibility to draw robust conclusion, this suggests this score alone was unable to explain the difference in clinical status. We thus investigated the impact of the rare genetic variation in constrained genes that were shared by affected individuals in multiplex families. Interestingly, the negative correlation that we observed between PRS and rare damaging variants specifically in unaffected individuals suggests that the balance between rare and common variants might explain the difference in clinical manifestation that we observed in multiplex families of BD. The small size of our sample forces us to consider these results with caution and replication on independent larger samples will be needed to confirm such hypothesis.

Genes with rare damaging mutations which were shared by affected subjects in multiplex families revealed a network of proteins involved in neuronal development or in the regulation of neuronal gene expression. These pathways have already been reported in numerous WES in ASD or SZ^7,8,39^, indicating a clear overlap in the etiology of BD with these disorders as already suggested^9–11,14^. These findings are also consistent with results from GWAS and transcriptome analyses of SZ and, to a lesser extent of ASD^6,43,44^, suggest that the regulation of gene expression during brain development can be a common pathogenic pathway impaired in BD, SZ, and ASD.

Here, we identified a set of four novel genes, for which the mutation rate in individuals with BD was significantly higher with respect to the general population. Among these genes, *SMARCC2* (MIM: 601734) encodes a scaffolding core subunit of the chromatin remodeling complex mSWI/SNF that controls the accessibility of DNA sequences to transcription factors. This complex plays an essential role during neuron development and the absence of *Smarcc2* in mice resulted in impaired embryonic and adult neurogenesis inducing cognitive dysfunction^45^. Mutations in this gene have already been found in patients with ASD^46^ or neurodevelopmental disorder^47^, and mutations in another subunit of this complex, SMARCA2, have been reported in individuals with SZ^48,49^.

*HELLS* (MIM: 603946), the second gene identified to be frequently mutated in patients with BD, belongs to the SNF2 subfamily of chromatin remodeling ATPase as SMARCA2. This protein is required for genome-wide methylation^50^ and it is responsible of immunodeficiency ventromeric region instability and facial anomalies (ICF) syndrome (MIM: 616910). The ICF syndrome is characterized by centromeric instability, as the cytogenetic hallmark, facial dysmorphism, and severe immunodeficiency, as well as developmental delay and intellectual deficit. In mice, HELLS was demonstrated as regulator of neural stem cell fate, affecting self-renewal and proliferation of neural progenitor cells^51^, suggesting a direct role in nervous system development.

The third gene frequently mutated in our cohort of individuals with BD is *UPF2* (MIM: 605529), a core component of the nonsense-mediated mRNA decay (NMD) pathway, a surveillance pathway that eliminates mRNA with premature translation termination codon. In drosophila, *Upf2* plays a role for proper development of synapse architecture and synaptic vesicle efficacy^52^. Moreover, copy number variants in this gene were found in intellectual disabilities syndrome with DiGeorge syndrome associated^53^ and de novo point mutations have been identified in a patient with SZ^54^. Altogether, these observations suggest a role for UPF2 in nervous system development.

Few things are known about the last gene, *WDR37*. Its protein belongs to the WD40 repeat domain family that is characterized by diverse cellular function, as chromatin assembly, RNA processing, immunity, or development^55^. This interacting domain scaffolds protein–protein or protein–DNA interactions. Although WDR37 function is unknown, mice knockout for this gene show a larger brain than wild-type littermates^56^. Interestingly, 57 copy number variants including this gene have been reported in the DECIPHER v9.30 released (https://decipher.sanger.ac.uk/). Among associated phenotypes, we reported developmental delay, seizures, intellectual disabilities, and ASD.

In summary, we demonstrated that both common and rare variants contribute to phenotypic outcome in multiplex families of BD, and our data suggest that the difference between the affected and unaffected status of individuals within multiplex families might come from the balance between the two types of variants. In addition, we confirmed the shared genetic vulnerability between BD, SZ, and ASD and suggest the familial forms of BD might share a higher heritability with SZ than non-familial ones. Finally, we highlighted a neurodevelopmental impairment in familial forms of BD. Altogether, our results shed new lights on the genetic architecture and biology of BD and highlight the importance of using large pedigrees with a high density of affected individuals to investigate the etiology of the disease.

## Supplementary information

Supplementary figures

Table S1

Table S2

Table S3

Table S4
