# Supplementary material for: Contribution of common and rare damaging variants in familial forms of bipolar disorder and phenotypic outcome
Source: Transl Psychiatry. 2020 Apr 28;10:124. doi: 10.1038/s41398-020-0783-0 (PMC7188882; doi:10.1038/s41398-020-0783-0)
Supplement: Supplementary file 1 — Supplementary figures [file 41398_2020_783_MOESM1_ESM.pdf]

Supplementary figures

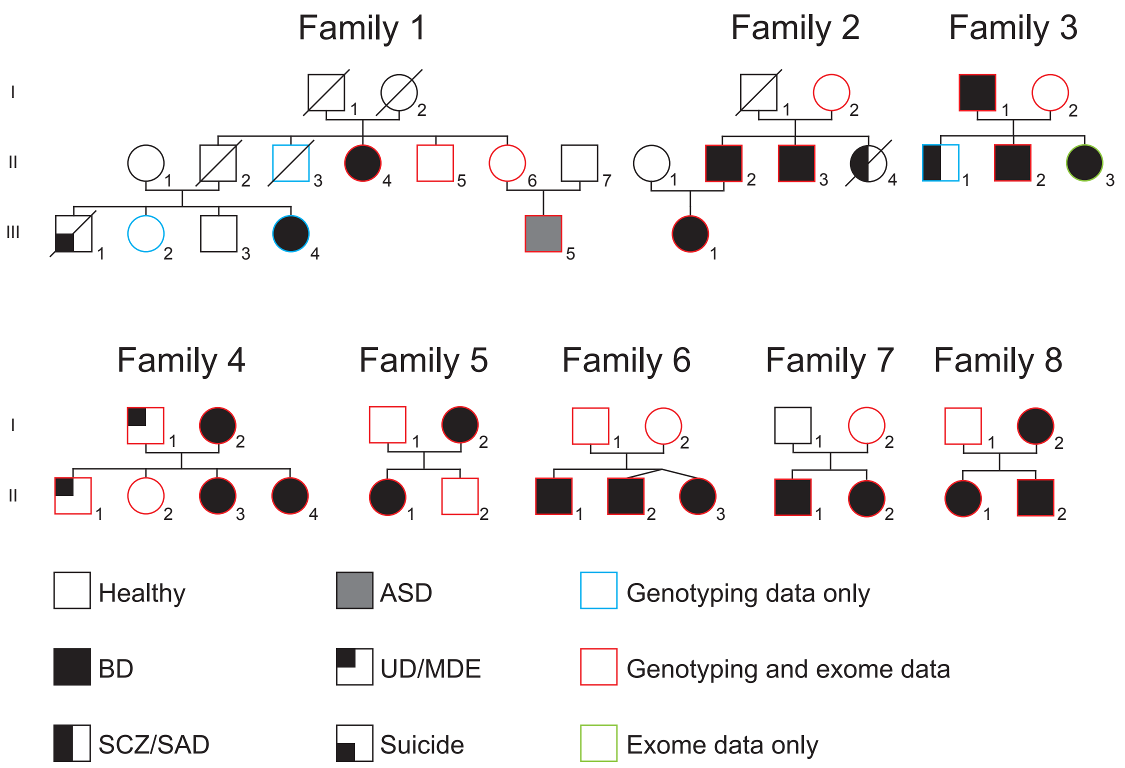

Fig. S1. Multiplex families with bipolar disorder.

Individual diagnoses are shown in black or grey, with black filled symbol for bipolar disorder (BD), black half-filled symbol for schizophrenia (SZ) or schizoaffective disorder (SAD), black up quarter-filled for unipolar depression (UD) or major depressive episode (MDE), black down quarter-filled for suicide and grey filled for autism spectrum disorder (ASD). Individuals for whom DNA has been genotyped and sequenced for the whole-exome are shown with red borders. Those for whom only whole-exome sequencing has been done are shown with green borders and those with blue borders have only been genotyped. The squares and the circles represent men and women respectively and slash marks show deceased individuals.

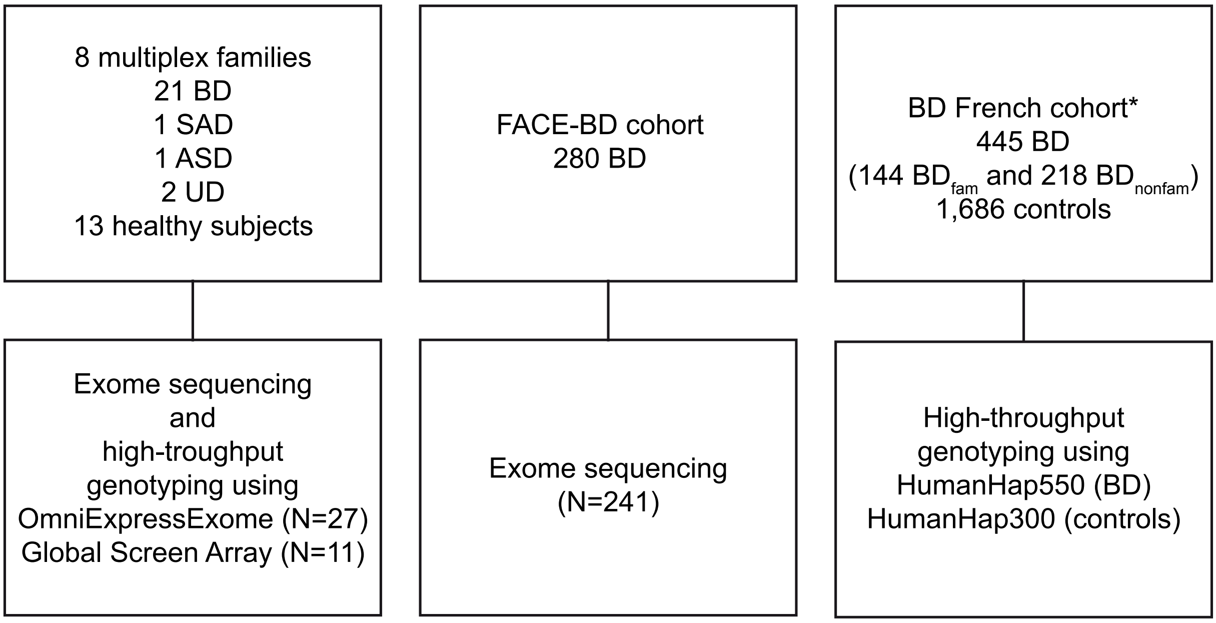

Fig. S2. Cohort description and their genetic analyses. ASD, individual with autism spectrum disorder; BD, individuals with bipolar disorder; FACE-BD, FondaMental Academic Centers of Expertise for Bipolar Disorder; SAD, individual with schizo-affective disorder; UD, individuals with unipolar depression or major depressive disorder. *this cohort and its genotyping data have been previously published.[7,33]

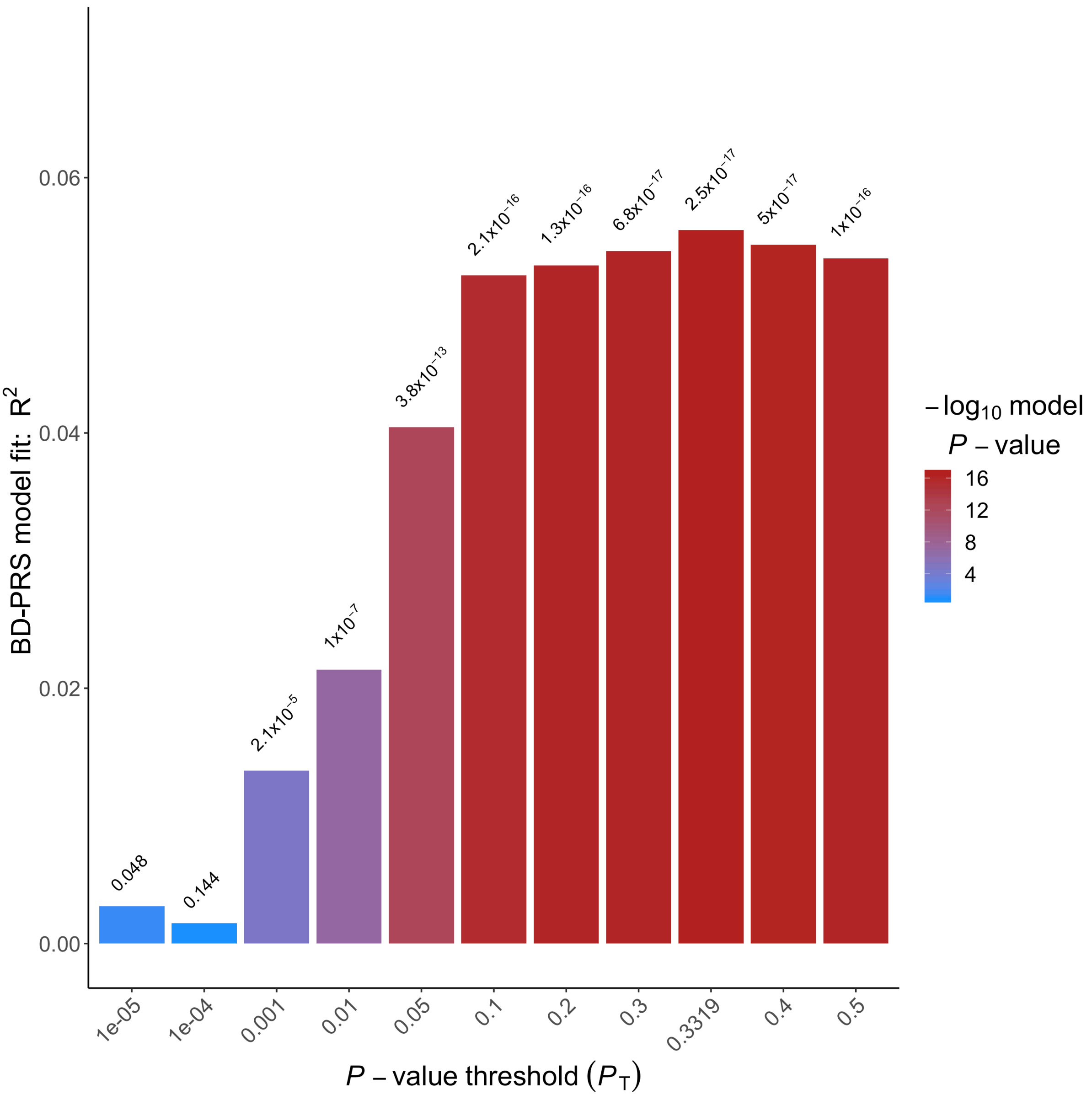

Fig. S3. Estimation of the best threshold p-value for the bipolar disorder polygenic risk score to discriminate individuals with bipolar disorder and controls. Bar plot shows the model fit and p-value association at different p-value thresholds for bipolar disorder polygenic risk score (BD-PRS).

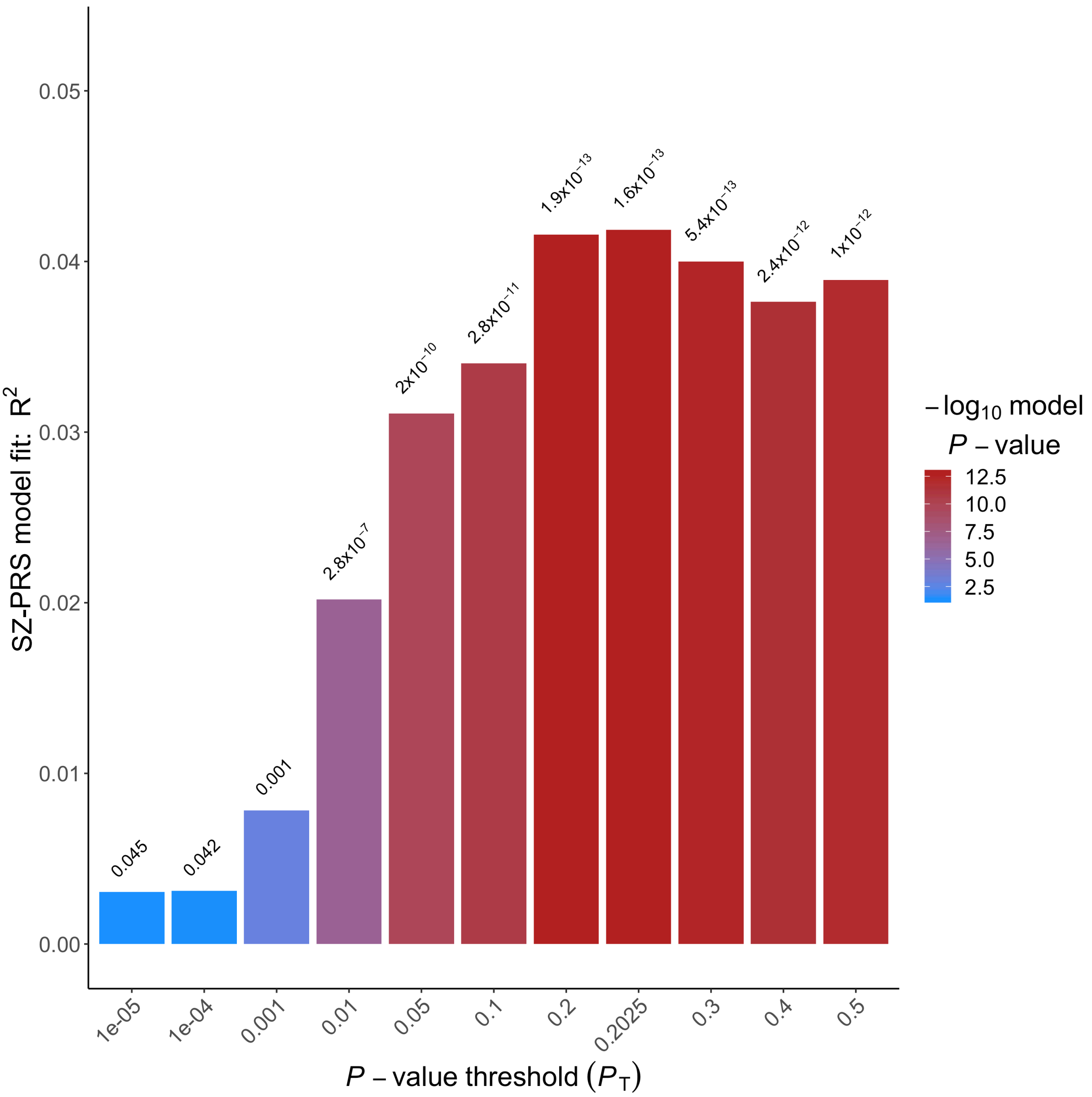

Fig. S4. Estimation of the best threshold p-value for the schizophrenia polygenic risk score to discriminate individuals with bipolar disorder and controls. Bar plot shows the model fit and p-value association at different p-value thresholds for schizophrenia polygenic risk score (SZ-PRS).

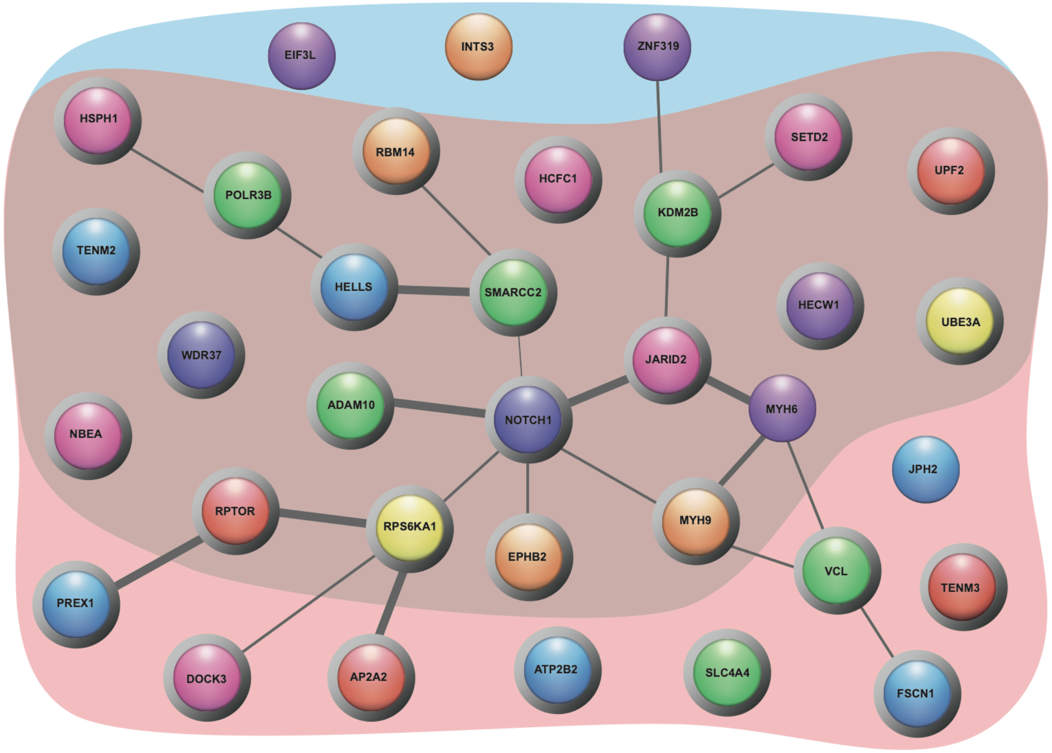

Fig. S5. Interaction network and biological pathways containing proteins encoded by the genes with damaging mutations segregating in affected subjects in multiplex families with bipolar disorder.

Network and pathways analyses have been conducted using a combination of results from the STRING software, a review of the literature and individual Gene Ontology terms. The lines show protein-protein interactions with a thickness proportional to the confidence of the interactions (0.4-0.6, 0.6-0.8, >0.8). Balls of the same color showed genes identified in a same family. Most of the genes were involved either in development and morphogenesis (red cloud) or in the regulation of gene expression (blue cloud). Grey circles show the 29 out of 34 genes that are implied in neuronal pathways.

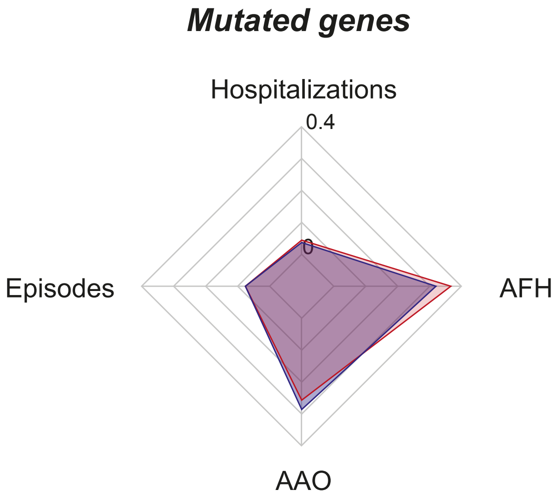

Fig. S6. Clinical severity of individuals mutated in *SMARCC2*, *HELLS*, *UPF2* and *WDR37*.

Radar plots represent the severity of the disease in individuals with (red) or without (blue) mutation in *SMARCC2*, *HELLS*, *UPF2* and *WDR37*. The disease severity was defined with the total number of hospitalizations and the total number of episodes (manic, hypomanic, mixed or depressive episode with or without psychotic features) by year of disease, the reciprocal of the age at onset (AAO) and the reciprocal of the age at first hospitalization (AFH). For graphical representation, we zoomed on the median values of normalized data for mutated and non-mutated individuals for each feature.
