# Supplementary material for: Contribution of common and rare damaging variants in familial forms of bipolar disorder and phenotypic outcome
Source: Transl Psychiatry. 2020 Apr 28;10:124. doi: 10.1038/s41398-020-0783-0 (PMC7188882; doi:10.1038/s41398-020-0783-0)
Supplement: Supplementary file 2 — Table S1 [file 41398_2020_783_MOESM2_ESM.docx]

**Table S1. List of damaging mutations identified in multiplex families and shared by affected subjects**

| **Chr** | **Position (bp)** | **A1** | **A2** | **SNP** | **Mutation type** | **Gene symbol** | **Transcript** | **Amino acid change** | **CADD** | **Family** | **Individuals**^a^ |
| --- | --- | --- | --- | --- | --- | --- | --- | --- | --- | --- | --- |
| 3 | 10401576 | C | T | rs61736451 | missense | *ATP2B2* | NM_001001331.2 | Val631Met | 22.9 | 1 | II.4, II.6, III.4 |
| 5 | 167689409 | G | A | rs370494324 | missense | *TENM2* | NM_001122679.1 | Arg2631His | 23.4 | 1 | II.4, II.5, II.6, III.4, III.5 |
| 7 | 5633129 | G | A | rs371640415 | missense | *FSCN1* | NM_003088.3 | Val188Met | 23.8 | 1 | II.3, II.4, II.6, III.4 |
| 10 | 96333792 | G | A | - | missense | *HELLS* | NM_001289067.1 | Asp185Asn | 18.95 | 1 | II.3, II.4, II.5, II.6, III.4, III.5 |
| 20 | 42747220 | C | T | rs557878787 | missense | *JPH2* | NM_020433.4 | Ala405Thr | 25.8 | 1 | II.3, II.4, II.6, III.4, III.5 |
| 20 | 47247325 | A | T | rs138500849 | missense | *PREX1* | NM_020820.3 | Tyr1512Asn | 26.9 | 1 | II.3, II.4, II.6, III.4, III.5 |
| 1 | 26872509 | G | A | rs762876090 | missense | *RPS6KA1* | NM_001006665.1 | Gly40Ser | 21.5 | 2 | I.2, II.2, II.3, III.1 |
| 15 | 25616068 | T | C | - | missense | *UBE3A* | NM_000462.3 | Lys421Arg | 22 | 2 | I.2, II.2, II.3, III.1 |
| 4 | 183714082 | G | A | rs753345698 | missense | *TENM3* | NM_001080477.3 | Arg2086His | 31 | 3 | I.1, II.1, II.2, II.3 |
| 10 | 11997377 | T | G | rs373340381 | missense | *UPF2* | NM_015542.3 | Asn902His | 23.6 | 3 | I.1, II.1, II.2, II.3 |
| 11 | 988619 | A | G | rs144441591 | missense | *AP2A2* | NM_001242837.1 | Asn401Ser | 24.6 | 3 | I.1, II.1, II.2, II.3 |
| 17 | 78896587 | G | A | rs144632265 | missense | *RPTOR* | NM_020761.2 | Ala862Thr | 22.1 | 3 | I.1, II.1, II.2, II.3 |
| 4 | 72399974 | C | T | rs140882617 | missense | *SLC4A4* | NM_001134742.1 | Pro771Ser | 19.07 | 4 | I.2, II.1, II.3, II.4 |
| 10 | 75856965 | C | A | - | missense | *VCL* | NM_014000.2 | Leu583Ile | 27.2 | 4 | I.2, II.2, II.3, II.4 |
| 12 | 56577976 | G | A | rs775317449 | missense | *SMARCC2* | NM_003075.3 | Pro182Leu | 26 | 4 | I.2, II.3, II.4 |
| 12 | 106821117 | T | C | rs199504211 | missense | *POLR3B* | NM_018082.5 | Met415Thr | 26.9 | 4 | I.2, II.2, II.3, II.4 |
| 12 | 121880448 | CTT | - | rs544442304 | inframe deletion | *KDM2B* | NM_032590.4 | Lys932del | 20.8 | 4 | I.2, II.2, II.3, II.4 |
| 15 | 58957340 | T | C | rs145518263 | missense | *ADAM10* | NM_001110.3 | Arg181Gly | 22.5 | 4 | I.2, II.3, II.4 |
| 1 | 23240163 | G | A | rs368971406 | missense | *EPHB2* | NM_001309193.1 | Ala990Thr | 16.27 | 5 | I.2, II.1, II.2 |
| 1 | 153745424 | C | T | rs774874092 | missense | *INTS3* | NM_001324475.1 | Ala973Val | 25.3 | 5 | I.2, II.1, II.2 |
| 11 | 66392825 | C | T | rs139642034 | missense | *RBM14* | NM_006328.3 | Ala493Val | 23.3 | 5 | I.2, II.1, II.2 |
| 22 | 36681918 | C | T | rs148109368 | missense | *MYH9* | NM_002473.5 | Gly1715Ser | 19.49 | 5 | I.2, II.1, II.2 |
| 9 | 139413120 | C | T | - | missense | *NOTCH1* | NM_017617.4 | Ser341Asn | 19.18 | 6 | I.2, II.1, II.2, II.3 |
| 10 | 1125960 | A | - | - | frameshift | *WDR37* | NM_014023.3 | Ile83fs | 34 | 6 | I.1, II.1, II.2, II.3 |
| 3 | 47164504 | C | T | rs144677816 | missense | *SETD2* | NM_014159.6 | Arg541Gln | 24.3 | 7 | I.2, II.1, II.2 |
| 3 | 51315051 | A | G | - | missense | *DOCK3* | NM_004947.4 | Met897Val | 17.74 | 7 | II.1, II.2 |
| 6 | 15501422 | G | A | rs142763537 | missense | *JARID2* | NM_004973.3 | Asp744Asn | 22.5 | 7 | I.2, II.1, II.2 |
| 13 | 31736120 | - | G | - | frameshift | *HSPH1* | NM_001286504.1 | Arg38fs | 22.9 | 7 | I.2, II.1, II.2 |
| 13 | 36239272 | G | T | rs41292207 | missense | *NBEA* | NM_015678.4 | Val2784Phe | 26.4 | 7 | I.2, II.1, II.2 |
| X | 153222831 | G | A | rs782341890 | missense | *HCFC1* | NM_005334.2 | Pro763Ser | 23.9 | 7 | I.2, II.1, II.2 |
| 7 | 43495992 | C | T | rs201757499 | missense | *HECW1* | NM_015052.4 | Pro866Leu | 23.7 | 8 | I.2, II.1, II.2 |
| 14 | 23874889 | C | T | rs140596256 | missense | *MYH6* | NM_002471.3 | Glu98Lys | 27.1 | 8 | I.2, II.1, II.2 |
| 16 | 58030656 | C | A | rs149758697 | missense | *ZNF319* | NM_020807.2 | Arg505Leu | 23.3 | 8 | I.2, II.1, II.2 |
| 22 | 38246066 | C | G | - | missense | *EIF3L* | NM_016091.3 | His26Gln | 24.7 | 8 | I.2, II.1, II.2 |

^a^Individual position refers to Fig. 1

A1, reference allele; A2, alternative allele; bp, base pairs; Chr, chromosome; SNP, single nucleotide polymorphism
